# The Dietary Inflammatory Index and Its Associations with Biomarkers of Nutrients with Antioxidant Potential, a Biomarker of Inflammation and Multiple Long-Term Conditions

**DOI:** 10.3390/antiox13080962

**Published:** 2024-08-08

**Authors:** Angela A. Mulligan, Marleen A. H. Lentjes, Jane Skinner, Ailsa A. Welch

**Affiliations:** 1 Centre for Population Health Research, Faculty of Health, University of East Anglia, Norwich NR4 7TJ, UK; maria.lentjes@oru.se (M.A.H.L.); jane.skinner@uea.ac.uk (J.S.); 2 School of Medical Sciences, Faculty of Medicine and Health, Örebro University, 70182 Örebro, Sweden

**Keywords:** multiple long-term conditions, MLTCs, multi-morbidity, MM, dietary inflammatory index, biomarker, validation, antioxidant

## Abstract

We aimed to validate the Dietary Inflammatory Index (DII^®^) and assess the cross-sectional associations between the DII^®^ and multiple long-term conditions (MLTCs) and biomarker concentrations and MLTCs using data from the European Prospective Investigation into Cancer (EPIC-Norfolk) study (11,113 men and 13,408 women). The development of MLTCs is associated with low-grade chronic inflammation, and ten self-reported conditions were selected for our MLTC score. Data from a validated FFQ were used to calculate energy-adjusted DII^®^ scores. High-sensitivity C-reactive protein (hs-CRP) and circulating vitamins A, C, E, β-carotene and magnesium were available. Micronutrient biomarker concentrations were significantly lower as the diet became more pro-inflammatory (*p*-trend < 0.001), and hs-CRP concentrations were significantly higher in men (*p*-trend = 0.006). A lower DII^®^ (anti-inflammatory) score was associated with 12–40% higher odds of MLTCs. Lower concentrations of vitamin C and higher concentrations of hs-CRP were associated with higher odds of MLTCs. The majority of the associations in our study between MLTCs, nutritional biomarkers, hs-CRP and the DII^®^ were as expected, indicating that the DII^®^ score has criterion validity. Despite this, a more anti-inflammatory diet was associated with higher odds of MLTCs, which was unexpected. Future studies are required to better understand the associations between MLTCs and the DII^®^.

## 1. Introduction

The Dietary Inflammatory Index (DII^®^) is a literature-based dietary score that was developed to measure the potential impact of diet on the inflammatory status of an individual [1]. The biological damage resulting from reactive oxygen species (ROS) is known as oxidative stress, which is induced by inflammation, and it results in a lowering of the antioxidant capacity of cells [2,3]. Diets rich in antioxidants, such as vitamins A, C and E, β-carotene, selenium, flavonoids and phytoestrogens, which are included in the DII^®^ score, may potentially play an important role in modulating inflammation [4]. Antioxidants offer protection against a number of chronic conditions [5], including cancer [6], depression [7], cardiovascular disease [8], stroke [9] hypertension [10,11], type 2 diabetes [12,13,14] and obesity [14].

The National Institute for Health and Care Excellence (NICE) defines multiple long-term conditions (MLTCs), or multi-morbidity (MM), as the presence of two or more long-term health conditions in an individual [15]. These conditions can include defined physical or mental health conditions, such as type 2 diabetes or schizophrenia; ongoing conditions, such as learning disability; symptom complexes, such as frailty or chronic pain; sensory impairment, such as sight or hearing loss; and alcohol or substance misuse [15]. However, there is currently no international consensus on how to define and measure MLTCs, but a number of recent reviews have attempted to progress towards reaching a more standardised approach [16,17,18].

The prevalence of MLTCs in ageing populations is increasing, leading to huge healthcare and personal costs. ‘Inflamm-aging’ is used to describe chronic low-grade inflammation that is characteristic of increasing age [19], which has been related to a number of chronic diseases, including cancer [20], cardiovascular disease [21], type 2 diabetes [22] and depression [23], that contribute to MLTCs. Research indicates that diets rich in antioxidants, such as β-carotene, vitamins A, C and E and magnesium, may play an important role in modulating inflammation. High-sensitivity C-reactive protein (hs-CRP) is a well-known inflammatory biomarker, and previous studies have reported that elevated concentrations of hs-CRP are associated with a higher risk of cancer and the incidence of other chronic diseases [20,24]. Lifestyle factors, such as diet, smoking and physical activity, can affect an individual’s state of systemic inflammation [25,26,27], which has been shown to promote the development of diseases such as cancer [20], cardiovascular disease [21], type 2 diabetes [22], musculoskeletal conditions [28] and depression [23].

Using data for England, in 2015, 54.0% of people aged 65 and over suffered from MLTCs; by 2035, this is predicted to have risen to 67.8% [29]. The authors estimate that there will be 17.0% of people aged 65 and over living with four or more conditions in 2035, compared with 9.8% in 2015; by disease, most people aged 65 and over will be affected by arthritis (62.6%), hypertension (55.9%), respiratory disease (24.4%), cancer (23.7%) and type 2 diabetes (21.6%) [29]. In 2019, the UK spent GBP 50.5 billion on related long-term chronic conditions [30], making chronic diseases one of the major socio-economic challenges of our time. There are numerous adverse consequences of MLTCs; people will die prematurely [31] and have more hospital admissions, which will be of a longer duration [32]. Having MLTCs has an enormous effect on an individual’s quality of life, tending to impact more on physical than mental health [33].

Given the relevance of inflammation to the development of MLTCs and the potential for a more antioxidant and anti-inflammatory diet to influence the onset and progression of MLTCs, research is lacking on the associations between the DII^®^ and prevalence or onset of MLTCs. Furthermore, whilst the DII^®^ has been validated or associated with circulating CRP concentrations [34], few studies have investigated the associations between clinical nutritional biomarkers of nutrient intake concurrently with the presence of MLTCs [35,36]. Additionally, as it is difficult to accurately measure dietary intake, we therefore chose to validate the DII^®^ score against available concentrations of nutritional biomarkers, which are also DII^®^ parameters, to establish criterion validity, as there is currently a paucity of data in this area.

Considering these findings, further exploration of the potential associations between the consumption of an inflammatory diet and MLTCs, supported by nutritional data and circulating CRP, are required. Therefore, using cross-sectional data, this study firstly aims to validate the DII^®^ score against available nutritional biomarkers and hs-CRP. A secondary aim is to assess the associations between the DII^®^ score and MLTCs, and a third aim is to investigate the associations between biomarker concentrations and MLTCs. We will additionally assess the associations between hs-CRP and nutritional biomarkers.

## 2. Materials and Methods

### 2.1. EPIC-Norfolk Study Design

The European Prospective Investigation into Cancer (EPIC) Norfolk study is part of the Europe-wide EPIC study, which has more than half a million participants from ten countries [37]. The EPIC-Norfolk cohort study was primarily set up to investigate diet and the risk of developing cancer, but its research interests widened to study additional lifestyle exposures and the causes of other chronic conditions and mortality [38].

### 2.2. Study Population

Men and women, aged between 39 and 79 years, were recruited from 35 general practitioners’ surgeries located in the Norfolk region of East Anglia from 1993 to 1997. As the vast majority of the UK population is registered with a general practitioner’s surgery through the National Health Service, the general practitioner’s age sex registers are an ideal population-sampling frame. The Norfolk District Health Authority Ethics Committee granted approval for the study (98CN01), and all participants provided written, informed consent, adhering to the Declaration of Helsinki.

### 2.3. Assessment of Dietary Intake and Supplement Use

Dietary intake at the baseline examination was assessed using a semi-quantitative food frequency questionnaire (FFQ) consisting of a food list of 130 lines with an additional question on milk intake at the back of the questionnaire. This FFQ is designed to capture the average daily intakes of foods and drinks during the previous year. The EPIC-Norfolk FFQ has been extensively validated in this study population [39,40,41]. The FFQ data were calculated for nutrient contribution using the FETA (FFQ EPIC Tool for Analysis) tool [42] based on our earlier in-house system, CAFÉ (Compositional Analyses from Frequency Estimates) [43]. Outliers in energy intake were identified by using the ratio of energy intake (EI) to the basal metabolic rate (BMR), where the BMR was calculated using sex-specific Henry equations [44]. Participants in the top and bottom 0.5% of the EI:BMR ratio were excluded, as were those with FFQs containing 10 or more missing answers.

Intakes of foods and drinks from the FFQ were combined into crude food groups (expressed in grams). The food groups consisted of alcoholic beverages; grains and cereal-based products; eggs; fats and oils; fish and fish products; meat, including products and dishes; milk and dairy products; non-alcoholic beverages; nuts and seeds; potatoes; soups and sauces; sugars, preserves and snacks; and fruits, vegetables and legumes.

Participants who answered ‘Yes’ to the following question in the FFQ were classified as supplement users: ‘Have you taken any vitamins, minerals, fish oils, fibre or other food supplements during the past year?’.

### 2.4. The Dietary Inflammatory Index (DII^®^)

This is a literature-derived, population-based DII^®^, whose purpose is to compare diverse populations based on the inflammatory potential of their diets [1]. Qualifying articles (N = 1943) were scored according to whether each dietary parameter increased (+1), decreased (−1) or had no (0) effect on six inflammatory biomarkers: IL-1β, IL-4, IL-6, IL-10, TNF-α and C-reactive protein. Articles were weighted by study characteristics, and using these weighted values, the pro- and anti-inflammatory fractions for each food parameter were calculated.

### 2.5. Creation of the DII^®^

**Figure 1:**
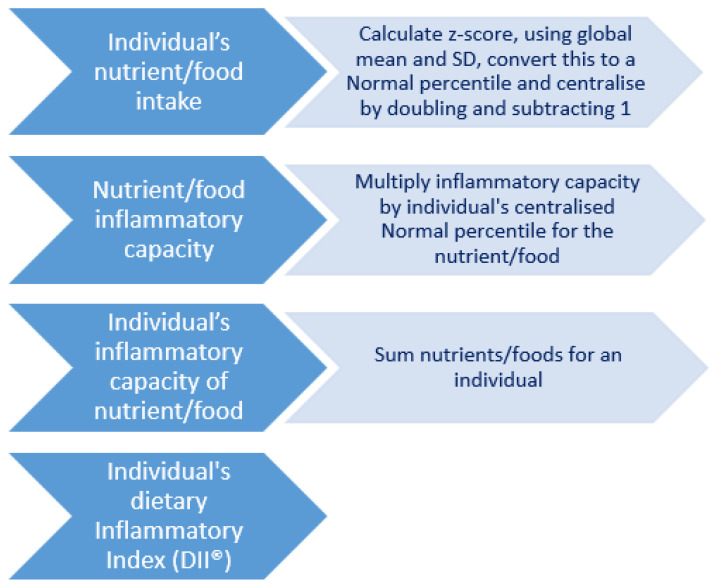
Steps in the creation of the DII^®^ score by Shivappa et al. [1].

Figure 1 illustrates the multi-step process required to create the DII^®^ score. The DII^®^ score was calculated using 37 dietary parameters. All of the pro-inflammatory parameters were included in the score: energy, carbohydrate, protein, total fat, saturated fat, trans fat, cholesterol, iron and vitamin B12. The anti-inflammatory parameters included alcohol, monounsaturated fatty acids (MUFAs), polyunsaturated fatty acids, (PUFAs), n-3 fatty acids, n-6 fatty acids, fibre, pyridoxine (B6), folic acid, riboflavin (B2), thiamine (B1), niacin, vitamins A, C, D and E, β-carotene, magnesium, selenium, zinc, flavan-3-ols, flavonols, flavones, anthocyanidins, isoflavones, pepper, onion, garlic and green/black tea. The residual method was used to obtain the energy-adjusted intakes for all nutrients. For the DII^®^ score calculation, dietary intakes were adjusted to a 2000 kcal/day diet to assess diet quality independently of diet quantity and to, in part, reduce measurement error, as energy intake is related to both under- and over-reporting of dietary intakes [45]. The most negative DII^®^ score implies the maximum anti-inflammatory diet, while the most positive score implies the maximum pro-inflammatory diet.

### 2.6. Blood Sample and Biomarker Analyses

A non-fasting blood sample was provided by 95% of participants at the baseline health examination. Blood was taken by venipuncture into plain and citrate monovettes. The blood was stored in a dark container overnight in a refrigerator at 4–7 °C and then spun at 2100× *g* for 15 min at 4 °C to obtain plasma and serum samples, which were stored at −196 °C.

Concentrations of vitamin A (retinol) and vitamin E, in the form of α-tocopherol, were available for a subset of the cohort (n = 6656) that consisted of previous nested case–control studies, where cases were defined by incident cardiovascular disease or cancer and four matched, disease-free controls [46]. Plasma concentrations were analysed at IARC, Lyon (France), using HPLC. Plasma vitamin E concentration was adjusted for cholesterol, as this is perceived to be a more reliable marker for vitamin E nutritional status [47,48]. The adjusted concentration is presented in µmol/mmol, calculated by dividing the plasma vitamin E concentration (µmol/L) by the total cholesterol concentration (mmol/L).

Plasma β-carotene concentration was available for 7495 participants selected from case–control studies nested within the EPIC-Norfolk study. Plasma samples were analysed for β-carotene concentrations by reversed-phase HPLC (HPLC-1100 system, Hewlett Packard) at IARC, Lyon (France), using a method based on that of Steghens et al. [49].

Concentrations of β-carotene and vitamins A and E were not used to investigate associations with MLTCs, as they came from nested case–control studies.

Approximately six months after the study had started, available funding enabled samples to be taken for vitamin C analysis using citrated plasma. Plasma for vitamin C was stabilised in a standardised volume of metaphosphoric acid, which was then stored at −70 °C. Plasma vitamin C concentration was determined using a fluorometric assay within one week of sampling [50].

Serum magnesium concentration was determined using blood samples that were prepared using a technique optimised for use in the EPIC study and stored in liquid nitrogen at −196 °C until analysed using an Olympus AU640 Chemistry Immuno Analyser (Quotient Bioresearch, Fordham, UK) to perform a xylidyl blue-based colorimetric assay (Beckman Coulter, Brea, CA, USA).

In 2008, previously frozen samples of serum collected were analysed for concentration of high-sensitivity CRP (hs-CRP) in 18,586 available samples using the AU640 Chemistry Immuno Analyser (Olympus Diagnostics, Watford, UK).

### 2.7. Calculation of the MLTC Score

Ten chronic conditions were selected to contribute to the MLTC score—myocardial infarction, stroke, type 2 diabetes, cancer, asthma, arthritis, depression, osteoporosis, hypertension and obesity—taking into account the most prevalent conditions included in the Quality and Outcomes Framework (QOF) of the UK General Practice [51]. The MLTC score was calculated by assigning one point for each condition, enabling a maximum score of ten.

Conditions were ascertained with the help of measurements taken (blood pressure, weight and height) or questionnaire data for the eight remaining conditions. At the baseline health examination, a trained nurse measured participants’ weight (to the nearest 0.1 kg) using digital scales (Salter, Oldham, UK). Height was measured (to the nearest 0.1 cm) using a free-standing stadiometer. Participants wore light clothing and no shoes for both measurements. Body mass index (BMI) was calculated as the body mass (weight) divided by the square of the height and is expressed in kg/m^2^. The body mass index (BMI) calculated using the measured height and weight at the baseline health examination was used to categorise the participants as underweight (<18.5 kg/m^2^), normal weight (≥18.5 to <25 kg/m^2^), overweight (≥25 to 30 kg/m^2^) or obese (≥30 kg/m^2^).

A trained nurse took two measurements of systolic and diastolic blood pressures using an Accutorr sphygmomanometer with participants in a seated position after having rested for three minutes. The most appropriate cuff size was selected to consider the arm circumference, and the mean of the two blood pressure readings was used in the analyses.

A self-administered health and lifestyle questionnaire (HLQ) before the baseline examination provided data on the prevalence of a number of conditions. Participants were asked about their medical histories with the question “Has the doctor ever told you that you have any of the following?”, followed by a list of conditions that included heart attack, stroke, type 2 diabetes, cancer, asthma, arthritis, depression, osteoporosis and hypertension. Where participants did not answer the question relating to any of the chronic conditions, it was assumed that they did not have the condition. The number of participants affected were as follows: heart attack (n = 31), stroke (n = 22), type 2 diabetes (n = 29), cancer (n = 22), asthma (n = 28), arthritis (n = 58), depression (n = 44) and osteoporosis (n = 50).

Participants were classified as having hypertension if they fulfilled any of the following criteria: measured systolic blood pressure ≥ 140 mmHg, measured diastolic blood pressure ≥ 90 mmHg, stated that the doctor had diagnosed them as having high blood pressure (hypertension) requiring treatment with drugs or reported taking anti-hypertensive medication [52].

We summed the number of chronic conditions per individual and created a binary variable, i.e., those with zero or one chronic condition and those with two or more conditions.

### 2.8. Measurement of Other Associated Variables

The HLQ, which was completed by participants just before the baseline examination, provided data to enable the categorisation of a number of variables. Social class at HLQ was defined using the Registrar General’s occupation-based classification system. Non-manual occupations were represented by the following codes: I (professional), II (managerial and technical) and IIIa (non-manual skilled), whilst the codes for manual occupations were as follows: IIIb (manual skilled), IV (partly skilled) and V (unskilled) [53]. In this paper, these five classes were categorised into two groups, manual and non-manual, with a ‘missing’ third group for those who did not answer the question.

Educational status was based on the highest qualification achieved, which was categorised into four groups: degree or equivalent, A level or equivalent, O level or equivalent and less than O level or no qualifications. In our analyses, those with an educational status of O level and above were combined into one category, and a ‘missing’ category was created for participants who did not answer the question.

Participants were categorised as either ‘current smokers’ if they currently smoked cigarettes, ‘former smokers’ if they were a smoker previously and ‘never smokers’ were those who had never smoked (derived from the HLQ). A ‘missing’ category was created for those who did not provide an answer to the question.

Usual physical activity was derived using data from questions in the HLQ, relating to occupational and recreational activity over the previous year. Using a simple index, participants were assigned to one of four groups: inactive, moderately inactive, moderately active and active [54,55,56].

### 2.9. Inclusion and Exclusion Criteria for Analysis

**Figure 2:**
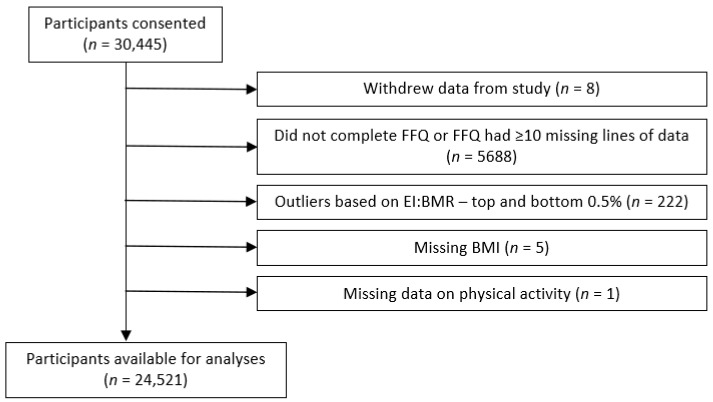
Study population included in analyses.

Figure 2 shows the numbers of participants available for analyses. In order to minimise data exclusions, missing data for a number of variables were treated in the following ways. A “missing category” was created for those with missing data on educational level, social class or smoking status (n = 16, 520 and 202, respectively). Data were available for analyses for 11,113 men and 13,408 women.

### 2.10. Statistical Analyses

**Figure 3:**
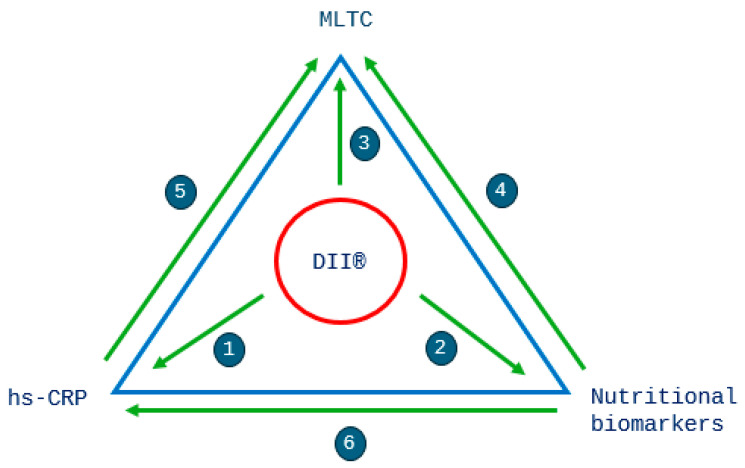
Overview of the research questions and analyses. The following associations were investigated: 1—DII^®^ and hs-CRP; 2—DII^®^ and nutritional biomarkers (vitamin C and Mg); 3—DII^®^ and MLTCs; 4—nutritional biomarkers and MLTCs (vitamin C and Mg); 5—hs-CRP and MLTCs; 6—hs-CRP and nutritional biomarkers (β-carotene, vitamins A, C and E and Mg).

All analyses were stratified by sex, as an independent *t*-test showed significant differences existed in the DII^®^ score (*p* < 0.001) between men and women. *p* < 0.05 was considered to be statistically significant in the analyses. The analyses were performed with the Stata statistical software version 17.0 (Stata Corp., College Station, TX, USA). Our analysis strategy is best observed in Figure 3.

#### 2.10.1. Descriptive Analyses

Descriptive statistics (means and SDs for continuous variables and frequencies and percentages for categorical variables) were analysed for all participants by sex-specific quintiles of the DII^®^ score, adjusted for a 2000 kcal diet. Linear regression and the chi-squared test for trends were used to test for trends for selected continuous and categorical variables, respectively, across sex-specific quintiles of the DII^®^ score. Where the percentage difference in biomarker concentrations between quintiles 1 and 5 is shown, this was calculated as Q5 − Q1/Q1 × 100. Reported intakes of crude food groups are described to assess their contribution over the DII^®^ spectrum. Food group data were not adjusted for energy intake.

#### 2.10.2. Associative Analyses

Binary logistic regression was used to determine odds ratios (ORs) of having two or more MLTCs (as opposed to zero or one) for quintiles 1 to 4 of the DII^®^ score (with quintile 5—most pro-inflammatory diet—as the reference category), using a series of cumulative adjustment models (Figure 3—research question 3). Model 1: unadjusted; model 2: adjusted for age; model 3: adjusted for age, smoking, physical activity, social class and educational level. ORs are presented with 95% confidence intervals (CIs). Trends in the results by DII^®^ quintile were calculated by replacing the quintile number with the median values of the DII^®^ within each quintile and modelling this as a continuous variable in the logistic regression [57].

To assess the association between the biomarkers and MLTCs, we used the same strategy as above (Figure 3—research questions 4 and 5). The highest concentrations (quintile 5) were used as the reference category. We assessed concentrations of hs-CRP and nutritional biomarkers across quintiles of the DII^®^ score (Figure 3—research questions 1 and 2). Additionally, we investigated associations between hs-CRP and nutritional biomarkers (Figure 3—research question 6).

## 3. Results

Not having any of the ten conditions included in the MLTC score was reported by 3052 (27%) men, 4376 (39%) reported having one condition and 3685 (33%) reported having two or more conditions (see Supplementary Figure S1). Not having any of the conditions was reported by 3437 (26%) women, while 4711 (35%) reported having one condition and 5260 (39%) reported having two or more conditions. The mean (SD) of chronic conditions in men was 1.18 (1.01), and in women, it was 1.32 (1.10). In this study, 2.1% of men and 3.4% of women were classified as having four or more of the ten conditions included in our MLTC score. More than 50% of men and more than 40% of women had hypertension (Supplementary Figure S2), with the second most common condition being arthritis in both men and women. In both men and women, more than 10% reported being obese, and more than 10% of women reported depression.

### 3.1. Characteristics of the Study Population

Selected characteristics of men (n = 11,113) and women (n = 13,408) by quintiles of the DII^®^ score, adjusted for a 2000 kcal diet, are shown in Table 1. The median DII^®^ scores were lower (i.e., less inflammatory) in women than in men.

Mean age, weight and BMI were significantly lower in men as the diet became more pro-inflammatory (*p*-trend < 0.001), whereas in women, only mean weight and BMI were significantly lower if the diet was more pro-inflammatory (*p*-trend < 0.001 and <0.05 respectively). Men and women whose diet was classified as the most anti-inflammatory (Q1) had the highest usage of supplements, with a significantly lower supplement consumption observed with the consumption of a more pro-inflammatory diet (*p* < 0.001). In both men and women, the percentage of manual workers, those who had no qualifications, current smokers and those who were physically inactive was significantly higher with a more pro-inflammatory diet (*p* < 0.001).

### 3.2. Food Group Consumption

Additionally, we studied the associations between quintiles of the DII^®^ score and the percentage contribution of weights of food groups. In both men and women, the intake of fruit, vegetables and legumes was lower with a more pro-inflammatory diet (see Supplementary Figure S3a,b). Intakes of milk and dairy products, non-alcoholic beverages, and sugars, preserves and snacks were generally higher as the diet was more pro-inflammatory. However, the gradients of intakes across the quintiles were generally small, with the greatest proportional differences between Q1 and Q5 observed for fruits, vegetables and legumes (−52% in men and −50% in women) and sugars, preserves and snacks (+136% in men and +180% in women).

### 3.3. Validation of the DII^®^ Score

We studied the associations between the DII^®^ score and directly measured inflammation (CRP) and nutritional biomarkers, vitamin C and magnesium, which were available for most of the study population, and β-carotene and vitamins A and E, available from nested case–control studies, to validate the obtained DII^®^ score. We chose antioxidant nutritional biomarkers previously associated with diet as well as disease risk.

Table 2 shows that, in men, the mean concentrations of β-carotene, vitamin A, cholesterol-adjusted vitamin E, magnesium and vitamin C were all generally lower as the diet became more pro-inflammatory (*p*-trend < 0.001). In women, the mean concentrations of β-carotene, vitamin A, cholesterol-adjusted vitamin E and vitamin C were lower as the diet became more pro-inflammatory (*p*-trend < 0.001), but not magnesium. In terms of inflammation, the hs-CRP concentrations were significantly higher in men with increasing DII^®^ quintile (*p*-trend = 0.006) but not in women (*p*-trend = 0.125).

### 3.4. Associations between the DII^®^ Score and MLTCs

Table 3 presents the results for the associative analyses between the DII^®^ and MLTCs. The percentages of people with a chronic condition did not vary greatly across quintiles of the DII^®^ score, nor did the percentages of each of the individual ten conditions (see Supplementary Figures S2 and S3, respectively).

In men, all three models indicated higher ORs of having two or more chronic conditions when the diet was more anti-inflammatory (*p*-trend < 0.001). The most anti-inflammatory diet had 45% higher odds than the most pro-inflammatory diet (model 1). The addition of age (model 2) slightly attenuated the associations in the lowest quintiles. Adjusting for additional factors (model 3) had minimal effect on the ORs.

For women, the unadjusted model 1 and model 2 showed no statistically significant associations for any quintile; also, the trend was non-significant (*p*-trend = 0.21 and 0.24, respectively). The addition of other covariates (model 3) increased the ORs proportionately more for the more anti-inflammatory diets, resulting in a significant trend (*p*-trend = 0.02).

Trend testing was achieved by replacing the quintile number with the median value of the DII^®^ score in the respective quintile.

### 3.5. Associations between Nutritional Biomarker Concentrations, Inflammation and MLTCs

**Figure 4:**
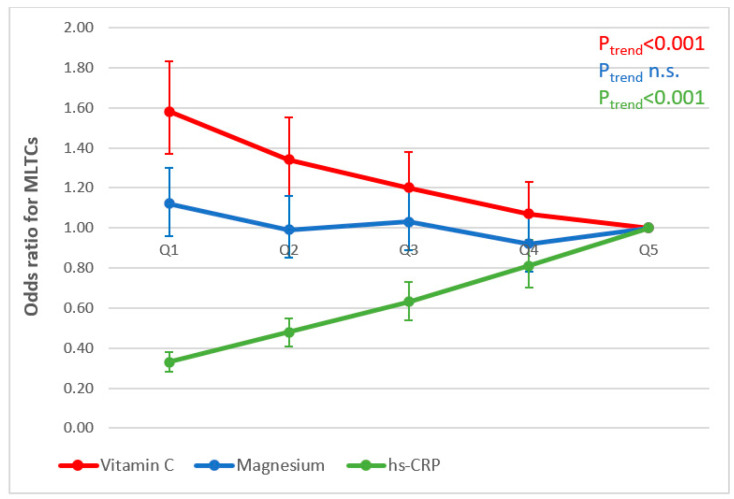
Associations between mean biomarker concentrations (quintiles, Q) and MLTCs in men (adjusted for age, smoking status, physical activity, educational level and social class).

**Figure 5:**
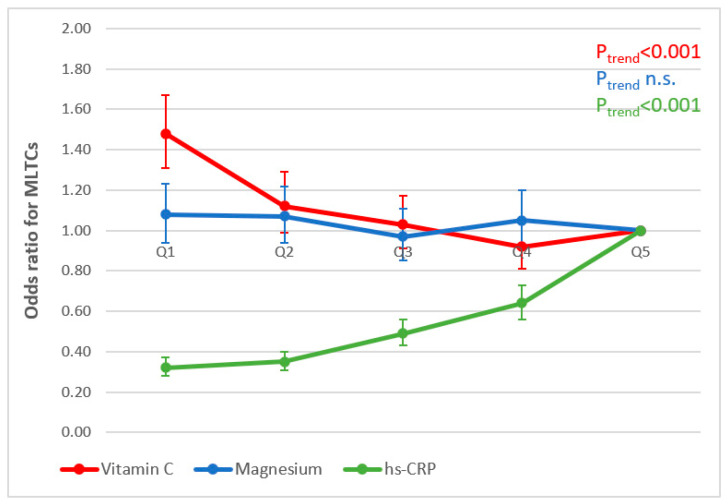
Associations between mean biomarker concentrations (quintiles, Q) and MLTCs in women (adjusted for age, smoking status, physical activity, educational level and social class).

Figure 4 and Figure 5 illustrate the associations observed between concentrations of nutritional biomarkers and inflammation and odds ratios of having MLTCs in men and women, respectively (research questions 2 and 3). Similar significant trends were observed for models 1 and 2; we therefore present the results for model 3 only (adjusted for age, smoking, physical activity, social class and educational level). In both men and women, higher ORs were observed of having two or more chronic conditions when concentrations of vitamin C were lower (*p*-trend < 0.001). In terms of inflammation, lower ORs of having two or more chronic conditions were associated with lower concentrations of hs-CRP (*p*-trend < 0.001) in both men and women. No significant associations were observed for magnesium in either men or women.

### 3.6. Associations between hs-CRP and Nutritional Biomarkers (Research Question 6)

For every standard deviation higher in the biomarker concentration, the hs-CRP concentrations were observed to be lower, with exception of magnesium and vitamin E, where the hs-CRP concentrations were observed to be higher (see Table 4).

## 4. Discussion

**Figure 6:**
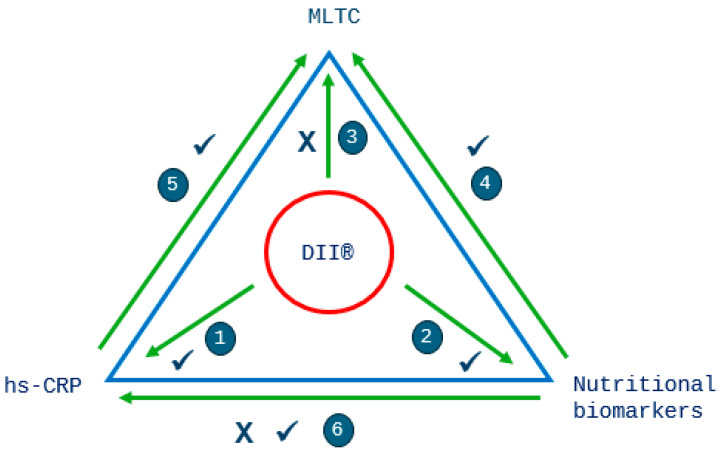
Summary of our findings in relation to the research questions, indicating expected and unexpected associations. Research questions: 1—DII^®^ and hs-CRP (Table 2); 2—DII^®^ and nutritional biomarkers (vitamin C and Mg) (Table 2); 3—DII^®^ and MLTCs (Table 3); 4—nutritional biomarkers and MLTCs (vitamin C and Mg) (Figure 4 and Figure 5); 5—hs-CRP and MLTCs (Figure 4 and Figure 5); 6—hs-CRP and nutritional biomarkers (β-carotene, vitamins A, C and E and Mg) (Table 4). A ✓ indicates an expected association and a X indicates an unexpected association.

We observed that a more pro-inflammatory diet was statistically significantly associated with higher hs-CRP, whilst circulating concentrations of β-carotene and vitamins A, C and E, anti-inflammatory and antioxidant vitamins, were lower. We also observed statistically significant higher ORs of having two or more chronic conditions when circulating concentrations of vitamin C were lower and lower ORs with lower concentrations of hs-CRP. Socio-economic and lifestyle factors, including social class, educational level, smoking status and physical activity, which are risk factors for chronic disease, were associated with the DII^®^ score in the direction that was expected. However, a more anti-inflammatory diet was associated with higher odds of MLTCs, which was the opposite from what we had hypothesised. The findings from this study (summarised in Figure 6), using direct measures of status of nutritional antioxidants, β-carotene and vitamins A, C and E, and directly measured CRP, indicate that the DII^®^ score has criterion validity for the inflammatory potential of diet in this population. However, the results relating to the DII^®^ score and ORs for having MLTCs warrant further scrutiny and may in part be explained by the cross-sectional design of our research. It is plausible that participants suffering from a chronic condition before the start of the study may have increased their consumption of certain foods such as fruits and vegetables, reflecting a more anti-inflammatory diet, which may lead one to incorrectly conclude that a more anti-inflammatory diet is associated with disease.

We observed that a diet with greater inflammatory potential (a higher DII^®^ score) was associated with higher hs-CRP concentrations. Results from cross-sectional studies on the association between the DII^®^ score and CRP have been mixed, but a recent systematic review and meta-analysis shows that higher DII^®^ scores are associated with a higher odds ratio of having raised plasma CRP levels [34]. Anti-inflammatory components of the DII^®^ include unsaturated fatty acids, vitamins and minerals, a number of which additionally have antioxidant properties, consumed in foods such as fruits, vegetables, legumes and wholegrains. These foods are also important components of other healthy dietary patterns, such as the Mediterranean Diet, which has been shown to be associated with lower CRP concentrations in cross-sectional studies [58]. Circulating concentrations of vitamin C are widely recognised as a valid biomarker for the consumption of fruits and vegetables [59], and our findings that a more anti-inflammatory DII^®^ score was associated with higher vitamin C concentrations and that lower concentrations of this antioxidant vitamin were related to higher MLTCs seem to support this. Data from the EPIC-Norfolk study have previously shown that higher concentrations of ascorbic acid (also when excluding supplement users) are associated with lower mortality [60]. Zhang et al. found that more frequent consumption of processed meat and poultry was associated with higher risks of MLTCs, whereas a higher intake frequency of total fish, fruits and cereal was associated with lower risks, in UK Biobank participants [61]. Less than 1% of total body magnesium is found in the blood, and under normal conditions, the body maintains tight homeostatic control of its concentration [62]. It is therefore unsurprising that we did not find any associations between magnesium concentrations and MLTCs. 

We are unaware of previous studies that have investigated the DII^®^ and MLTCs or direct measures of nutrient and inflammatory status (scoping review in preparation [63]), making our findings an important contribution to the literature on MLTCs, inflammation and diet. Ruel et al. found that a high consumption of fruit and vegetables and grain products other than rice and wheat could prevent the development of MLTCs in the Chinese population [64]. Protective associations have been found for higher fruit consumption and MLTCs in two cross-sectional studies in South Korea [65] and China [66]. Diets high in red meat and chicken were found to be among the main risk factors for MLTCs in middle-aged Australians [67]. Our observation that associations between the DII^®^ score and MLTCs were non-significant or in the opposite direction from what we expected (when associations between biomarkers and MLTCs were observed in the hypothesised directions) may have several reasons. Firstly, although the DII^®^ score parameters were classified into anti-inflammatory (e.g., vitamins A, C and E) or pro-inflammatory (e.g., saturated fats) [1], nutrients are seldom eaten in isolation. Moreover, the balance between the included nutrients and foods in the DII^®^ score does not represent the balance in daily dietary habits. Secondly, dietary assessment methods, especially FFQs, are known for misreporting, thereby impacting on nutrient intake and potentially misrepresenting the proportions between food groups included in the DII^®^ [68]. It is possible that there is a lack of capacity for the DII^®^ score, measured using an FFQ in this population, to appropriately assess associations with MLTCs.

The major strengths of our study include its large population of community-living, middle-aged and elderly men and women and the availability of information on a large number of directly measured or self-reported chronic conditions that comprise the presence of MLTCs as well as factors associated with MLTCs, including age, smoking habit, physical activity, social class and education. The availability of concurrent direct measures of nutrition (β-carotene and vitamins A, C and E and magnesium) and inflammation are also a strength. Objectively measured height and weight at the same time-point, to enable the classification of obesity, are also an advantage. The capacity to establish criterion validity for relationships between the DII^®^, biochemistry and socio-economic factors are also a major strength.

The main limitations of our research include the self-reported measures for a number of variables, including dietary intake, physical activity and disease history (from which we obtained the MLTC score). The self-reporting of chronic conditions lacked information on date of diagnosis, and time at risk for MLTCs could therefore not be assessed. Moreover, reverse causality may have played a role in our findings. Although dietary and anthropometric assessments, blood sampling and questions on medical history were collected concurrently, the absence of the date of onset of chronic conditions may have resulted in reverse causality. Participants may have changed their diet because they were unwell prior to entry to the study [69]. For example, participants suffering from a chronic condition some time before the study started may have increased their consumption of foods such as fruits, vegetables and fish, reflecting a more anti-inflammatory and antioxidant diet, which may lead to the incorrect conclusion that a more anti-inflammatory diet is associated with disease [69]. It is well established that participants who enrol in cohort studies are less likely to be disabled or seriously unwell, and this may impact the generalisability of our results [70]. Nevertheless, the data from the baseline examination show that this cohort was comparable to the UK national population for a number of characteristics, including age, sex and anthropometric measurements, but the cohort did have a lower percentage of current smokers [71].

In our study, the conditions contributing to the MLTC score were myocardial infarction, stroke, type 2 diabetes, cancer, asthma, depression, arthritis, osteoporosis, hypertension and obesity. We were unable to include certain conditions, such as chronic kidney disease, as this was not asked about in the HLQ. Since a large number of individuals had existing hypertension, arthritis or depression, this may have dominated our MLTC score. Seven of the conditions are a sub-set of the eleven that Diederichs et al. recommend should be included in MLTC indices [72]. Moreover, many of the most prevalent conditions listed in the Quality and Outcomes Framework (QOF) of the UK General Practice were included in the HLQ and thereby counted towards the MLTC score [51]. Dodds et al. recently compared the prevalence of MLTCs, defined using two-count and two-index approaches, using UK Biobank data and found a higher prevalence using the count than the index methods [73]. A recent study by MacRae et al. used English primary care data to investigate the impact of varying the conditions considered when measuring MLTCs [74] and recommend that researchers should consider using existing condition lists that are associated with the highest prevalence of MLTCs to enable comparisons across studies [75,76,77]. However, these researchers acknowledge that data availability may influence condition choice [74].

Although the DII^®^ developed by Shivappa et al. [1] includes 45 food parameters, only 37 food parameters were included in our study. However, the missing food parameters likely make up a small proportion of the total nutrients consumed within our study population (e.g., eugenol, ginger, rosemary, saffron, turmeric), and despite these missing parameters, we did observe associations with diet-related biomarkers. Findings from a previous study have validated the association between the DII^®^ score and circulating inflammatory marker concentrations, even when the number of available food parameters is limited [78]. Our data included three of the four flavonoid parameters, although previous research has shown that tea and fruits are the highest contributors to flavonoid intake in the UK, which are included in the score, either as a specific parameter or through a number of the vitamin components [79]. Isoflavone intake data were also included in the DII^®^ score, even though intake in the EPIC-Norfolk population is low and therefore unlikely to have made an important contribution to the overall score [80].

Research has shown how inflammation may contribute to the development of a number of chronic conditions. Dysfunction of the endothelium, induced by inflammation, has been associated with CVD and hypertension [81] and has also been linked to the development of insulin resistance and type 2 diabetes [82,83]. Infections have been estimated to be responsible for approximately 15% of cancers worldwide via a number of mechanisms, including chronic inflammation [84]. There is also evidence that inflammasome-mediated pathways may be associated with depression, cognitive decline and dementia, including Alzheimer’s disease [85]. A recent review found that age-related oxidative stress is potentially a contributing factor to the progression of a number of diseases, including CVD, neurodegenerative diseases, cancer and arthritis [86]. The consumption of an anti-inflammatory diet rich in antioxidants would therefore seem beneficial.

Future research in this area should ensure that the presence of MLTCs is clearly defined, preferably using a more established and accepted consensus. Data on when a chronic condition was first diagnosed, in relation to the period of dietary data collection, must be available. More high-quality analyses are required to add to the limited evidence on this topic.

## 5. Conclusions

We found that higher inflammation, measured by direct measurements of hs-CRP, and lower concentrations of the antioxidant nutrients β-carotene and vitamins A, C and E were consistently significantly associated with higher odds of having MLTCs. Given this, our findings that a more anti-inflammatory diet was associated with higher odds of MLTCs were unexpected given the associations we found with biochemical and nutritional biomarkers. Possible explanations lie in the complexity of dietary habits and inter-relationships between nutrients not covered in the DII^®^ score as well as methodological issues. However, based on the results from our analyses on biomarkers of diet and inflammation risk, the findings from our study show that the DII^®^ score has criterion validity for the inflammatory potential of the diet in this population of middle-aged and old men and women. Future studies require better and concurrent capture of the individual conditions comprising MLTCs, as well as more discriminating methods for defining MLTCs, in addition to direct biomarkers of inflammation, in order to unravel how the anti-inflammatory potential of diet may help in preventing diseases of ageing.

## Figures and Tables

**Table 1:** Selected characteristics of men and women by quintiles of the DII^®^ score.

	Quintile 1: Most Anti-Inflammatory	Quintile 2	Quintile 3	Quintile 4	Quintile 5: Most Pro-Inflammatory	*p* Trend
**MEN**	n = 2223	n = 2223	n = 2222	n = 2223	n = 2222	
DII^®^ range	−6.76 to −1.31	−1.31 to −0.02	−0.02 to 1.03	1.03 to 2.15	2.15 to 7.60	
DII^®^(median)	−2.24	−0.62	0.50	1.55	2.95	
MLTCs (n, %)	839 (38)	768 (35)	710 (32)	713 (32)	655 (29)	
Age (years)	60.5 (9.0)	60.1 (9.3)	59.4 (9.2)	59.5 (9.4)	58.9 (9.5)	<0.001
Weight (kg)	80.9 (11.2)	80.7 (11.4)	80.7 (11.2)	80.0 (11.4)	79.0 (11.5)	<0.001
BMI (kg/m^2^)	26.6 (3.3)	26.6 (3.3)	26.6 (3.2)	26.5 (3.3)	26.2 (3.2)	<0.001
Supplement user, %	50	42	38	32	29	<0.001
**Social class, %**						<0.001
Non-manual	65	61	58	55	48	
Manual	33	37	40	44	49	
Missing	2	2	2	1	2	
**Education, %**						<0.001
No qualifications	25	27	29	32	38	
O level and above	75	73	71	68	61	
Missing	0	0	0	0	0	
**Smoking status, %**						<0.001
Current	5	7	11	15	22	
Former	58	58	55	53	48	
Never	36	35	34	32	30	
Missing	1	0	1	1	1	
**Physical activity, %**						<0.001
Inactive	27	28	31	34	33	
Moderately inactive	26	26	26	25	20	
Moderately active	24	23	23	22	24	
Active	24	22	20	20	23	
**BMI, %**						0.012
Underweight	0	0	0	0	0	
Normal weight	31	32	31	32	36	
Overweight	56	54	55	54	52	
Obese	14	14	13	13	12	
	**Quintile 1: Most Anti-Inflammatory**	**Quintile 2**	**Quintile 3**	**Quintile 4**	**Quintile 5: Most Pro-Inflammatory**	***p*** **Trend**
**WOMEN**	n = 2682	n = 2682	n = 2681	n = 2682	n = 2681	
DII^®^ range	−6.62 to −2.17	−2.17 to −1.02	−1.02 to 0.01	0.01 to 1.18	1.18 to 6.71	
DII^®^ (median)	−2.95	−1.55	−0.50	0.55	2.08	
MLTCs (n, %)	1081 (40)	1073 (40)	1018 (38)	1045 (39)	1043 (39)	
Age (years)	59.1 (9.1)	58.8 (9.2)	58.7 (9.2)	58.7 (9.3)	58.9 (9.6)	0.569
Weight (kg)	68.6 (12.0)	68.1 (11.6)	67.8 (11.1)	68.1 (12.1)	67.1 (11.8)	<0.001
BMI (kg/m^2^)	26.3 (4.3)	26.2 (4.3)	26.1 (4.0)	26.3 (4.5)	26.0 (4.4)	0.001
Supplement user, %	64	58	53	49	42	<0.001
**Social class, %**						<0.001
Non-manual	65	64	60	58	55	
Manual	32	34	38	39	42	
Missing	2	2	2	3	3	
**Education, %**						<0.001
No qualifications	35	39	40	45	50	
O level and above	65	61	60	55	50	
Missing	0	0	0	0	0	
**Smoking status, %**						<0.001
Current	6	7	9	13	20	
Former	36	34	32	30	29	
Never	58	58	58	56	50	
Missing	1	1	1	1	1	
**Physical activity, %**						<0.001
Inactive	25	27	30	32	36	
Moderately inactive	31	33	34	34	30	
Moderately active	24	24	22	21	21	
Active	21	16	14	13	13	
**BMI, %**						0.005
Underweight	1	1	0	1	1	
Normal weight	42	43	43	43	46	
Overweight	41	40	41	38	37	
Obese	17	17	16	18	16	

Values are mean ± SD unless specified otherwise. *p*-value for trend for continuous variables was calculated using linear regression; chi-squared test for trend was used for categorical variables (missing category excluded from trend analyses).

**Table 2:** Biomarker concentrations for men and women by quintiles of the DII^®^ score.

	Quintile 1: Most Anti-Inflammatory	Quintile 2	Quintile 3	Quintile 4	Quintile 5: Most Pro-Inflammatory	Q5–Q1 Diff	% Diff	*p* Trend
**MEN**	n = 2223	n = 2223	n = 2222	n = 2223	n = 2222			
hs-CRP (nmol/L)	27.4 (51.2) (n = 1603)	27.7 (50.4) (n = 1609)	26.5 (45.2) (n = 1590)	27.5 (54.0) (n = 1622)	33.1 (72.7) (n = 1610)	5.7	20.9	0.006
β-carotene (µmol/L)	0.42 (0.25) (n = 761)	0.39 (0.25) (n = 737)	0.35 (0.22) (n = 727)	0.33 (0.18) (n = 741)	0.30 (0.18) (n = 707)	−0.12	−28.6	<0.001
Vitamin A (µmol/L)	1.87 (0.44) (n = 761)	1.86 (0.45) (n = 737)	1.84 (0.43) (n = 727)	1.82 (0.46) (n = 741)	1.77 (0.43) (n = 707)	−0.1	−5.4	<0.001
Vitamin E, adjusted for cholesterol (μmol/mmol)	4.56 (1.18) (n = 755)	4.42 (1.13) (n = 723)	4.39 (0.98) (n = 714)	4.31 (1.00) (n = 734)	4.02 (0.92) (n = 697)	−0.55	−12.0	<0.001
Vitamin C (µmol/L)	54.3 (17.5) (n = 1993)	50.9 (17.7) (n = 1973)	47.5 (17.6) (n = 1967)	44.2 (18.6) (n = 1981)	38.7 (18.8) (n = 1952)	−15.6	−28.8	<0.001
Magnesium (mmol/L)	0.82 (0.12) (n = 1602)	0.81 (0.12) (n = 1608)	0.82 (0.12) (n = 1591)	0.81 (0.12) (n = 1617)	0.81 (0.12) (n = 1611)	−0.001	−0.15	<0.001
**WOMEN**	n = 2682	n = 2682	n = 2681	n = 2682	n = 2681			
hs-CRP (nmol/L)	27.5 (48.2) (n = 1960)	29.4 (65.2) (n = 1997)	27.7 (56.5) (n = 2003)	30.7 (66.2) (n = 1976)	31.7 (62.5) (n = 1925)	4.2	15.2	0.125
β-carotene (µmol/L)	0.59 (0.35) (n = 693)	0.49 (0.27) (n = 696)	0.48 (0.29) (n = 723)	0.44 (0.28) (n = 704)	0.40 (0.23) (n = 701)	−0.19	−32.4	<0.001
Vitamin A (µmol/L)	1.78 (0.44) (n = 693)	1.80 (0.48) (n = 696)	1.75 (0.42) (n = 723)	1.72 (0.45) (n = 704)	1.70 (0.42) (n = 701)	−0.08	−4.3	<0.001
Vitamin E, adjusted for cholesterol (μmol/mmol)	4.64 (1.11) (n = 690)	4.50 (1.09) (n = 687)	4.45 (1.05) (n = 711)	4.37 (1.02) (n = 699)	4.14 (0.97) (n = 689)	−0.51	−10.9	<0.001
Vitamin C (µmol/L)	65.4 (18.6) (n = 2360)	62.1 (18.3) (n = 2351)	59.8 (18.4) (n = 2363)	57.0 (19.4) (n = 2323)	49.6 (21.2) (n = 2305)	−15.9	−24.3	<0.001
Magnesium (mmol/L)	0.79 (0.13) (n = 1959)	0.80 (0.12) (n = 1990)	0.80 (0.12) (n = 1998)	0.80 (0.12) (n = 1966)	0.80 (0.12) (n = 1920)	0.002	0.2	0.610

Values are mean ± SD. %diff = (Q5 − Q1)/Q1 × 100. *p*-value for trend for continuous variables was calculated using linear regression.

**Table 3:** Odds ratios of having MLTCs by quintiles of the DII^®^ score in men and women.

	Q1 (Most Anti-Inflammatory)		Q2		Q3		Q4		Q5 (Most Pro-Inflammatory)	
	OR	95% CI	OR	95% CI	OR	95% CI	OR	95% CI	OR	*p* Trend
**MEN** (n = 11,113)										
Model 1	1.45	1.28–1.64	1.26	1.11–1.43	1.12	0.99–1.28	1.13	0.99–1.28	1.00	<0.001
Model 2	1.35	1.19–1.54	1.20	1.05–1.36	1.10	0.96–1.25	1.10	0.96–1.25	1.00	<0.001
Model 3	1.40	1.23–1.60	1.22	1.07–1.39	1.11	0.97–1.26	1.09	0.95–1.24	1.00	<0.001
**WOMEN** (n = 13,408)										
Model 1	1.06	0.95–1.18	1.05	0.94–1.17	0.96	0.86–1.07	1.00	0.90–1.12	1.00	0.209
Model 2	1.06	0.94–1.19	1.06	0.95–1.19	0.97	0.87–1.09	1.02	0.91–1.14	1.00	0.243
Model 3	1.12	1.00–1.26	1.10	0.98–1.24	1.00	0.89–1.12	1.03	0.92–1.16	1.00	0.024

The outcome is having two or more chronic conditions. Q5 (most pro-inflammatory diet) is the reference category for the exposure. Model 1—unadjusted; model 2—adjusted for age; model 3—adjusted for age, smoking status, physical activity, educational level and social class.

**Table 4:** Regression of hs-CRP on nutritional biomarkers *.

	MEN					WOMEN			
	N	Mean (SD)	Exp (Coeff)	95% CI		N	Mean (SD)	Exp (Coeff)	95% CI
β-carotene	2767	19.4 (12.1)	0.81	0.78–0.84		2642	25.5 (15.8)	0.75	0.72–0.78
Vitamin A	2767	52.6 (12.8)	0.90	0.87–0.94		2642	49.9 (12.4)	1.04	0.99–1.08
Vitamin C	7728	46.9 (18.6)	0.80	0.78–0.82		9499	58.7 (19.8)	0.81	0.79–0.83
Vitamin E	2725	4.35 (1.02)	1.07	1.02–1.12		2612	4.34 (1.06)	1.05	1.01–1.09
Magnesium	7678	0.81 (0.12)	1.17	1.14–1.20		9430	0.80 (0.12)	1.16	1.13–1.19

* hs-CRP was log-transformed. The nutritional biomarker concentrations were divided by their standard deviation. The value of 0.81 exp(coeff) for β-carotene in men, for example, can thus be interpreted as a 19% fall in the geometric mean of hs-CRP with a one-SD increase in β-carotene.

## Data Availability

The authors will make the dataset available under a Data Transfer Agreement to any bona fide researcher who wishes to obtain the dataset in order to undertake a replication analysis. Although the dataset is anonymised, the breadth of the data included and the multiplicity of variables that are included in this analysis file as primary variables or confounding factors means that provision of the dataset to other researchers without a Data Transfer Agreement would constitute a risk. Requests for data sharing/access should be submitted to the EPIC Management Committee (epic-norfolk@mrc-epid.cam.ac.uk).

## Supplementary Materials

The following supporting information can be downloaded at: https://www.mdpi.com/article/10.3390/antiox13080962/s1, Supplementary Figure S1. Percentages of men (A) and women (B) who reported having zero, one or two or more chronic conditions by quintiles of the DII^®^ score; Supplementary Figure S2. Percentage of men (A) and women (B), who reported having any of the ten conditions, by quintiles of the DII^®^ score; Supplementary Figure S3a. Percentage contribution of weights of food groups to total weight of food and drinks by quintiles of the DII^®^ score in men; Supplementary Figure S3b. Percentage contribution of weights of food groups to total weight of food and drinks by quintiles of the DII^®^ score in women.
