# Supplementary material for: The Dietary Inflammatory Index and Its Associations with Biomarkers of Nutrients with Antioxidant Potential, a Biomarker of Inflammation and Multiple Long-Term Conditions
Source: Antioxidants (Basel). 2024 Aug 8;13(8):962. doi: 10.3390/antiox13080962 (PMC11351935; doi:10.3390/antiox13080962)
Supplement: Supplementary file 1 [file antioxidants-13-00962-s001.zip › antioxidants-2868423-supplementary.pdf]

A   B

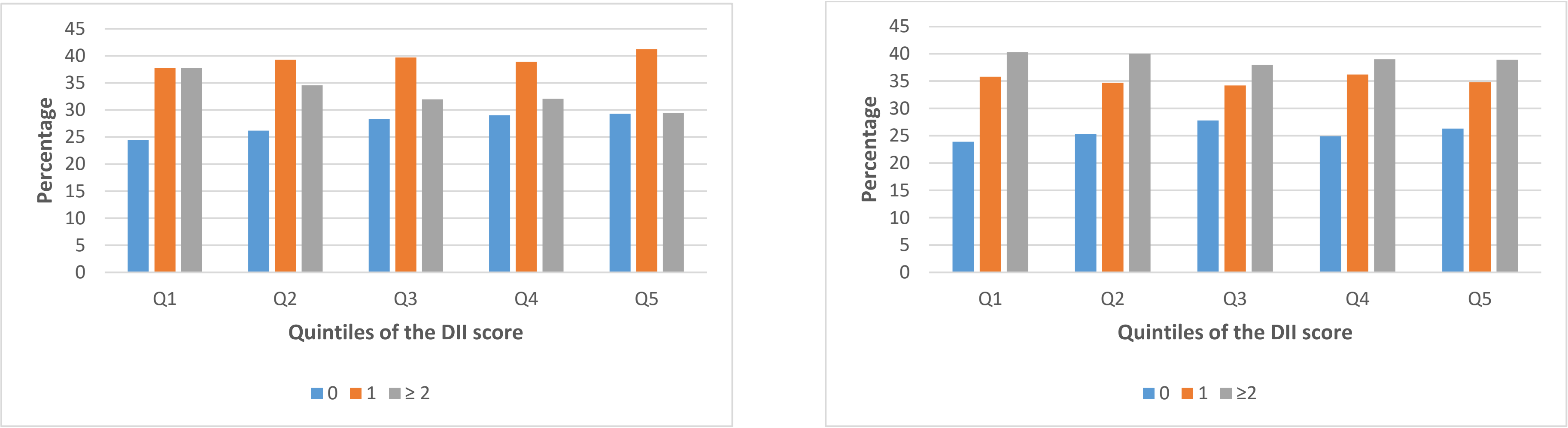

Supplementary Figure S1. Percentages of men (A) and women (B) who reported having zero, one, or two or more chronic conditions by quintiles of the DII® score (a higher quintile category indicates a more pro-inflammatory diet).

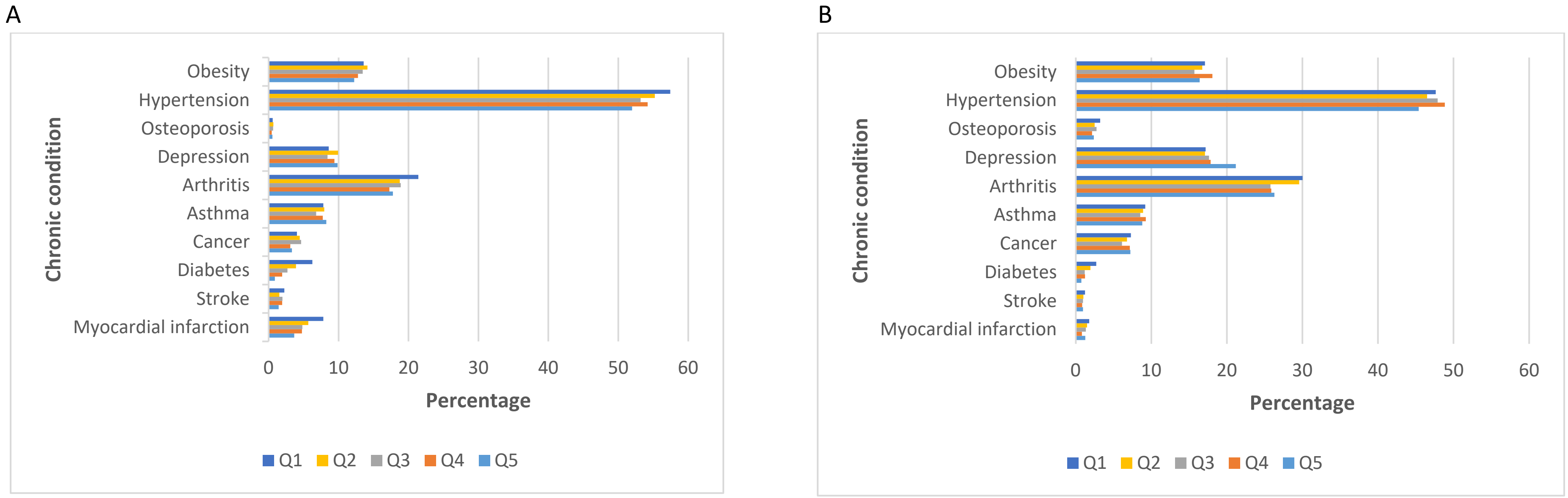

Supplementary Figure S2. Percentage of men (A) and women (B), who reported having any of the ten conditions, by quintiles of the DII® score (a higher quintile category indicates a more pro-inflammatory diet).

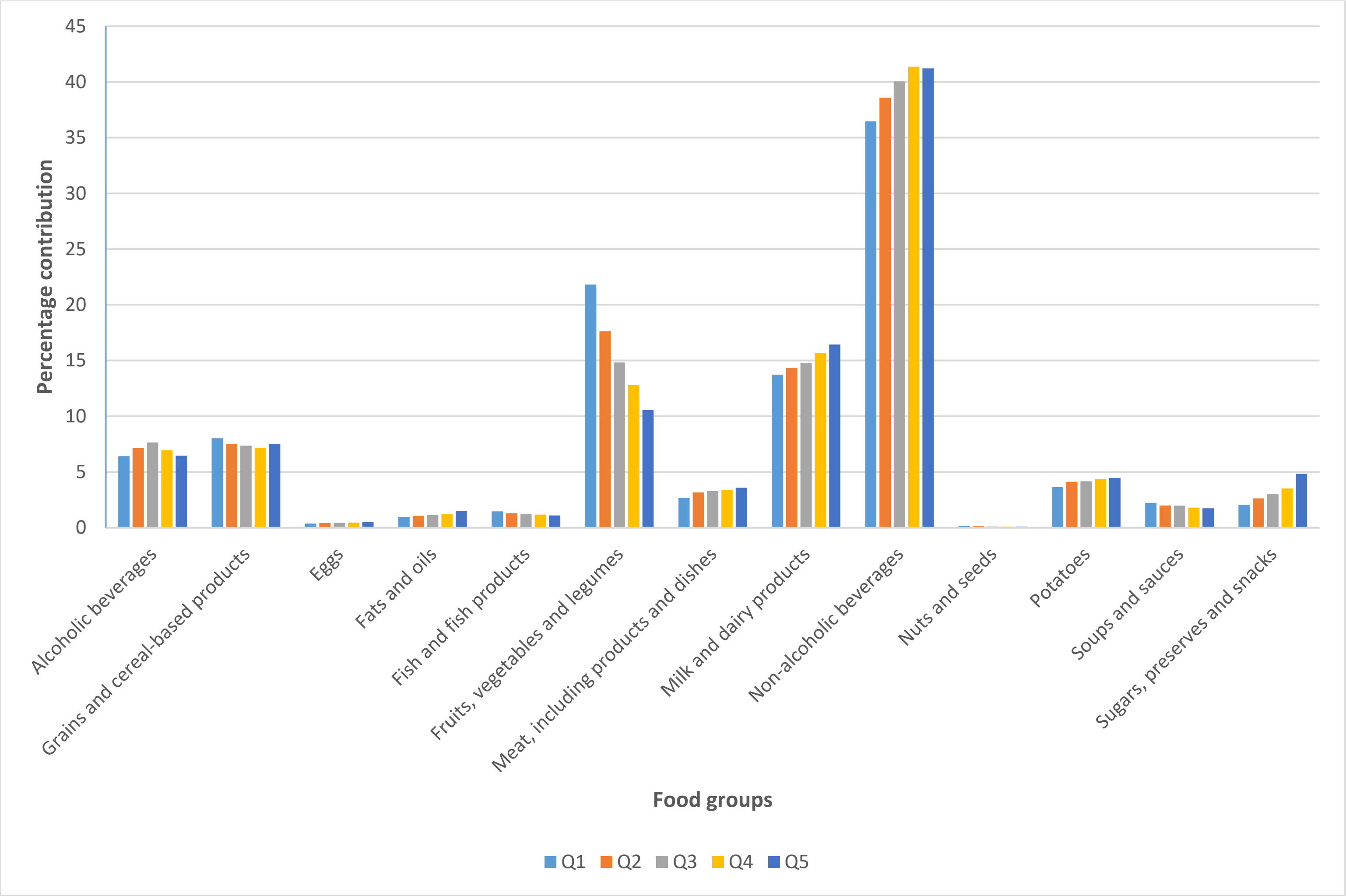

Supplementary Figure S3a. Percentage contribution of weights of food groups to total reported weight of food and drinks by quintiles of the DII® score in men (a higher quintile category indicates a more pro-inflammatory diet).

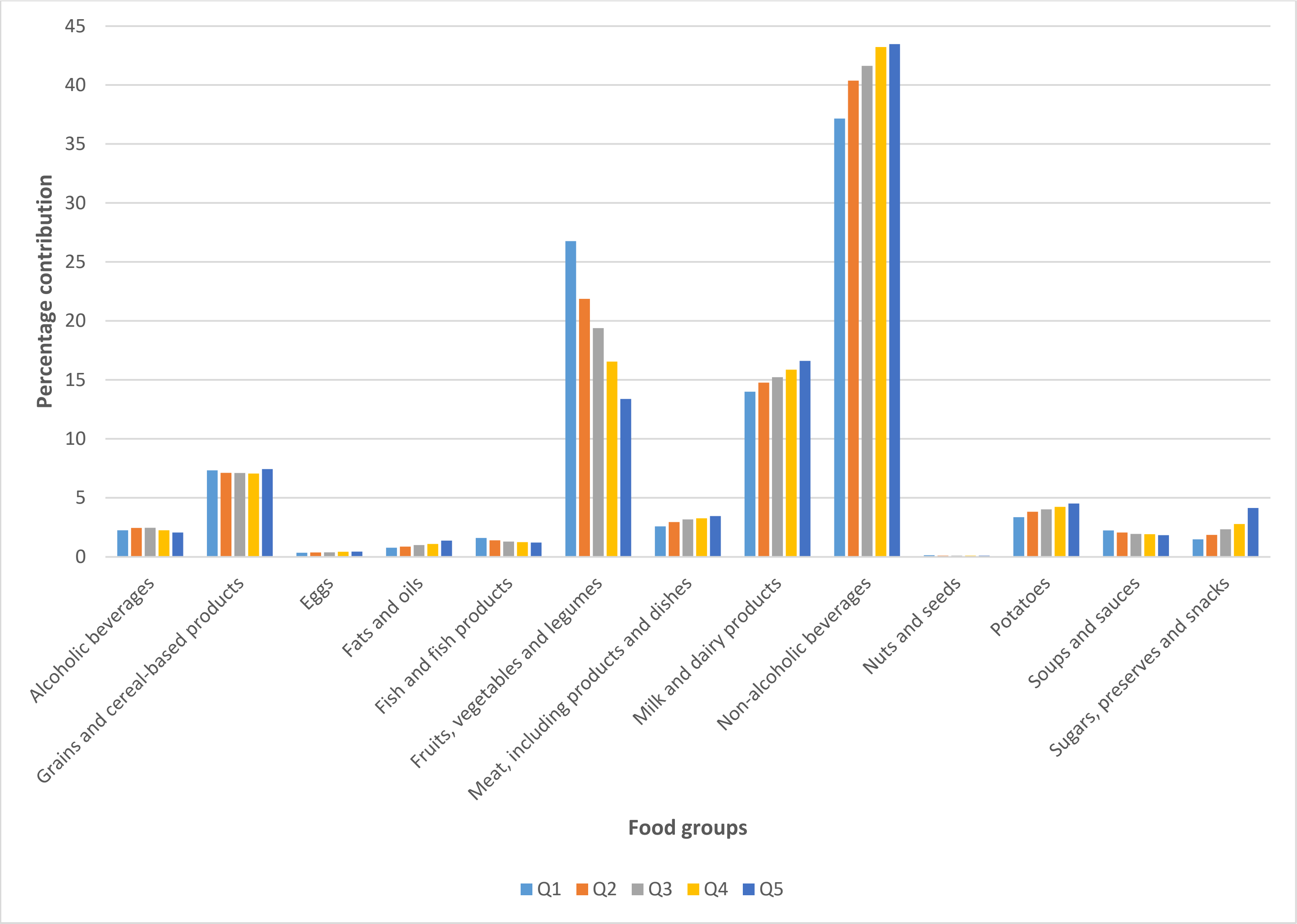

Supplementary Figure S3b. Percentage contribution of weights of food groups to total reported weight of food and drinks by quintiles of the DII® score in women (a higher quintile category indicates a more pro-inflammatory diet).
